# Regional transarterial chemoembolization combined with chemoradiotherapy for locally advanced rectal cancer: a retrospective study of a new combination

**DOI:** 10.3389/fonc.2023.1201544

**Published:** 2023-06-29

**Authors:** Wen-Jun Meng, Chun-Hua Liu, Ru-Jun Zheng, Chun-Xue Li

**Affiliations:** ^1^ Department of Biotherapy, Cancer Center, West China Hospital, Sichuan University, Chengdu, China; ^2^ Department of General Surgery, Daping Hospital, Army Medical University, Chongqing, China

**Keywords:** transarterial chemoembolization, neoadjuvant therapy, locally advanced rectal cancer, distant metastasis, prognosis

## Abstract

**Objectives:**

Locally advanced rectal cancer (LARC) has a high risk of distant metastasis (DM). Currently, many treatment courses of LARC have arisen, but patients’ DM status has not significantly improved. This study was designed to compare the effect between preoperative regional transarterial chemoembolization combined with neoadjuvant chemoradiotherapy and standard neoadjuvant therapy on preventing DM in patients with LARC.

**Methods:**

A total of 81 LARC patients between July 2013 and May 2018 were enrolled in this retrospective study. Among them, 44 patients received preoperative regional transarterial chemoembolization combined with concurrent chemoradiotherapy (the interventional group), and 37 patients received only neoadjuvant chemoradiotherapy (the control group). The baseline data; preoperative toxicities; postoperative DM rate within 1, 2, and 3 years; and postoperative complications were compared between the two groups.

**Results:**

All patients successfully completed their treatments. There were no significant differences between the two groups in age, gender, tumor size, distance between the tumor and anal verge, CEA level, lymphovascular invasion, or tumor stage before treatment. The pathological T staging post-treatment in the interventional group was significantly reduced compared to that of the control group (*p* = 0.025). There were no significant differences between groups in DM rates within 1 and 2 years after surgery. In terms of DM rate within 3 years after surgery, the interventional group was significantly lower than that of the control group (9.1% *vs.* 29.7%, *p* = 0.036).

**Conclusion:**

Preoperative regional transarterial chemoembolization combined with concurrent chemoradiotherapy may play an important role in reducing postoperative DM in LARC.

## Introduction

Rectal cancer is one of the most common and fatal malignant tumors in China and even around the world. Currently, the number of patients with rectal cancer is increasingly high. According to GLOBOCAN 2020, there were 732,210 new cases and 339,022 new deaths (1). Among the reasons for the treatment failure of rectal cancer, distant metastasis (DM) is the major one, of which the liver is the most common metastatic organ (2). According to recent published statistics, approximately 15% to 20% of rectal cancer patients are diagnosed with liver metastasis at their first diagnosis (3). Additionally, if no accurate or effective treatment is given, at least half of those premetastatic patients will ultimately develop rectal liver metastasis (RLM) during the treatment process (4). Thus, a worse quality of life and poorer prognosis might occur accordingly. Once RLM occurs, the 5-year survival rate is only approximately 10% (5). Locally advanced rectal cancer (LARC) is of middle-advanced stage, and has a higher risk of DM because of its treatment difficulty and rapid progression (6). Therefore, for LARC patients, apart from surgical operation, prevention of DM is also the focus during treatment. For this reason, neoadjuvant therapy has been proposed for years, but it only reduces the local recurrence rate of LARC. Among the current standard treatments, regardless of how the neoadjuvant chemotherapy regimen is adjusted or improved, patients’ long-term efficacy, especially DM rate, has not been significantly improved (7). Therefore, many creative regimens based on neoadjuvant therapy have been proposed such as total neoadjuvant therapy (TNT). For TNT, there also have not been any consensus because different conclusions have been reached in main large clinical trials (8, 9). As a result, despite the adjustment in the treatment course, patients’ DM has not significantly improved.

Preoperative regional transarterial chemoembolization, as a neoadjuvant and conversion therapy, has been successfully applied in liver and gastric cancer, but is rarely reported in the rectum (10, 11). Owing to the lack of large-scale clinical studies and guideline consensus, the method for rectal cancer was only implemented in a limited number of hospitals. Neoadjuvant chemotherapy for rectal cancer is based on 5-fluorouracil. However, whether the application of oxaliplatin can benefit patients in neoadjuvant therapy remains controversial. The CAO/ARO/AIO-04 study is currently the only large-scale study with positive results, with only improved disease-free survival (DFS) in LARC patients (12). Some studies even found that the addition of oxaliplatin increased the toxicity reactions with grade 3 to 4 among patients, which was not worth the gain (13). Based on the above, we designed this study by changing the route of administration of oxaliplatin. Under radioactive intervention, oxaliplatin was directly perfused into the tumor supplying arteries and supplemented by embolization. Since July 2013, this method has been successively used to treat primary tumors in LARC patients in our hospital, and has achieved good results. Now, our team continues to retrospectively compare whether it is different between this method combined with standard neoadjuvant therapy and traditional neoadjuvant therapy in preventing DM.

## Materials and methods

### Study design

From July 2013 to May 2018, a total of 81 patients with LARC who were admitted to our hospital were included in this retrospective cohort study. All patients had a complete record of at least 3 years of follow-up. The deadline for follow-up is May 2021. Among them, 44 patients received preoperative regional transarterial chemoembolization combined with neoadjuvant chemoradiotherapy (the interventional group), and 37 patients received only neoadjuvant chemoradiotherapy (the control group). This research was approved by the Ethics Committee of Daping Hospital of Army Medical University. All patients signed the relevant informed consent.

Patients’ inclusion criteria were (a) aged 18 to 75 years old; (b) the diagnosis of rectal adenocarcinoma was confirmed by the pathological biopsy performed before the treatment; (c) in accordance with the diagnostic criteria of LARC (clinical stage II or III); (d) patients have complete details of follow-up after hospitalization; and (e) performance status of 0–2 on the Eastern Cooperative Oncology Group (ECOG) criteria.

The exclusion criteria were (a) a history of abdominal surgery, radiotherapy, or chemotherapy; (b) medically uncontrolled serious heart, renal, or liver failure, hemorrhagic peptic ulcer, paralysis of intestine, ileus, or poorly controlled diabetes; (c) serious psyche or intelligence problem; (d) withdrawn halfway anytime during treatment or changed the established treatment plan without permission; and (e) incomplete medical records in our Computerized Patient Record System.

### Treatment regimen

**Figure 1:**
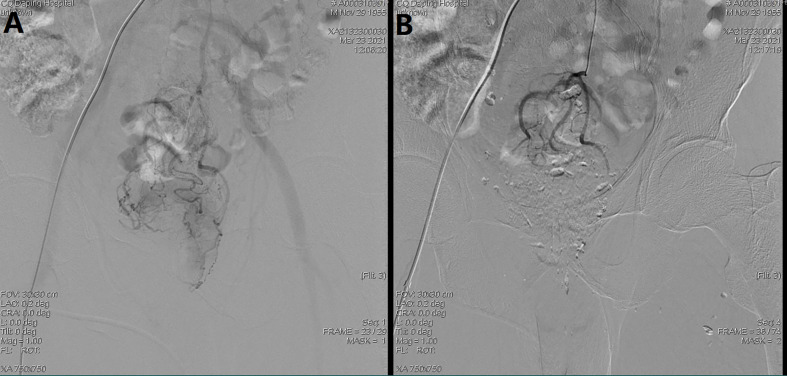
The images of a patient with locally advanced rectal cancer undergoing regional transarterial chemoembolization. **(A)** Iodixanol angiography in the inferior mesenteric artery. **(B)** chemoembolization for the postsuperior rectal artery completed.

Among the interventional group, the preoperative regional transarterial chemoembolization was performed on the first day. The detailed process of this method was as follows. First, the Seldinger method was used to puncture the femoral artery, and the catheter was sent into the inferior mesenteric artery under digital subtraction angiography (DSA). Iodixanol was chosen to serve as contrast medium to show the rectal tumor supplying arteries, and then a microcatheter was inserted through the catheter under imaging. Next, oxaliplatin (Jiangsu Hengrui Medicine Co., Ltd.) and normal saline were diluted to 50 ml, and infused slowly for regional chemotherapy for at least 10 min. The dosage of oxaliplatin was based on the patients’ body surface area (BSA; 85 mg/m^2^). After infusion chemotherapy, gelatin sponge particles (350–560 μm; Hangzhou Alicon) and iodixanol were injected into the superior rectal arteries for embolization. The chemoembolization process is shown with the representative images in **Figure 1**. Afterwards, long-course chemoradiotherapy was performed according to the guidelines of the National Comprehensive Cancer Network (NCCN). The oral chemotherapeutic agent was S-1 (Taiho Pharmaceutical Company) taken for four consecutive weeks, and the dosage was based on BSA (BSA < 1.25 m^2^, 80 mg; BSA = 1.25–1.5 m^2^, 100 mg; BSA > 1.5 m^2^, 120 mg).

Among the control group, long-course chemoradiotherapy was also performed according to the guidelines of the NCCN. The oral agent was capecitabine (Shanghai Roche Pharmaceutical Co., Ltd.), and its dosage was 825 mg/m^2^ in line with BSA, twice a day, 5 days a week, and taken for five consecutive weeks. Both groups received the same regimen of long-course irradiation. The total dosage was up to 50.4 Gy, 1.8 Gy/time, 5 days per week, then stopped for 2 days, and lasted for about five consecutive weeks.

All patients in both groups underwent laparoscopic surgical operation (Dixon or Miles) about 4 to 8 weeks after the end of neoadjuvant therapy. The surgical procedure was in accordance with the principle of total mesorectal excision. Among patients with sphincter preservation, a protective stoma of the ileum or sigmoid was routinely performed. XELOX or mFOLFOX6 adjuvant chemotherapy was continued for 4 to 6 months guided by the NCCN after radical surgery. Patients with protective stoma also underwent stoma closure 4 to 6 months after ostomy.

### Evaluation

**Figure 2:**
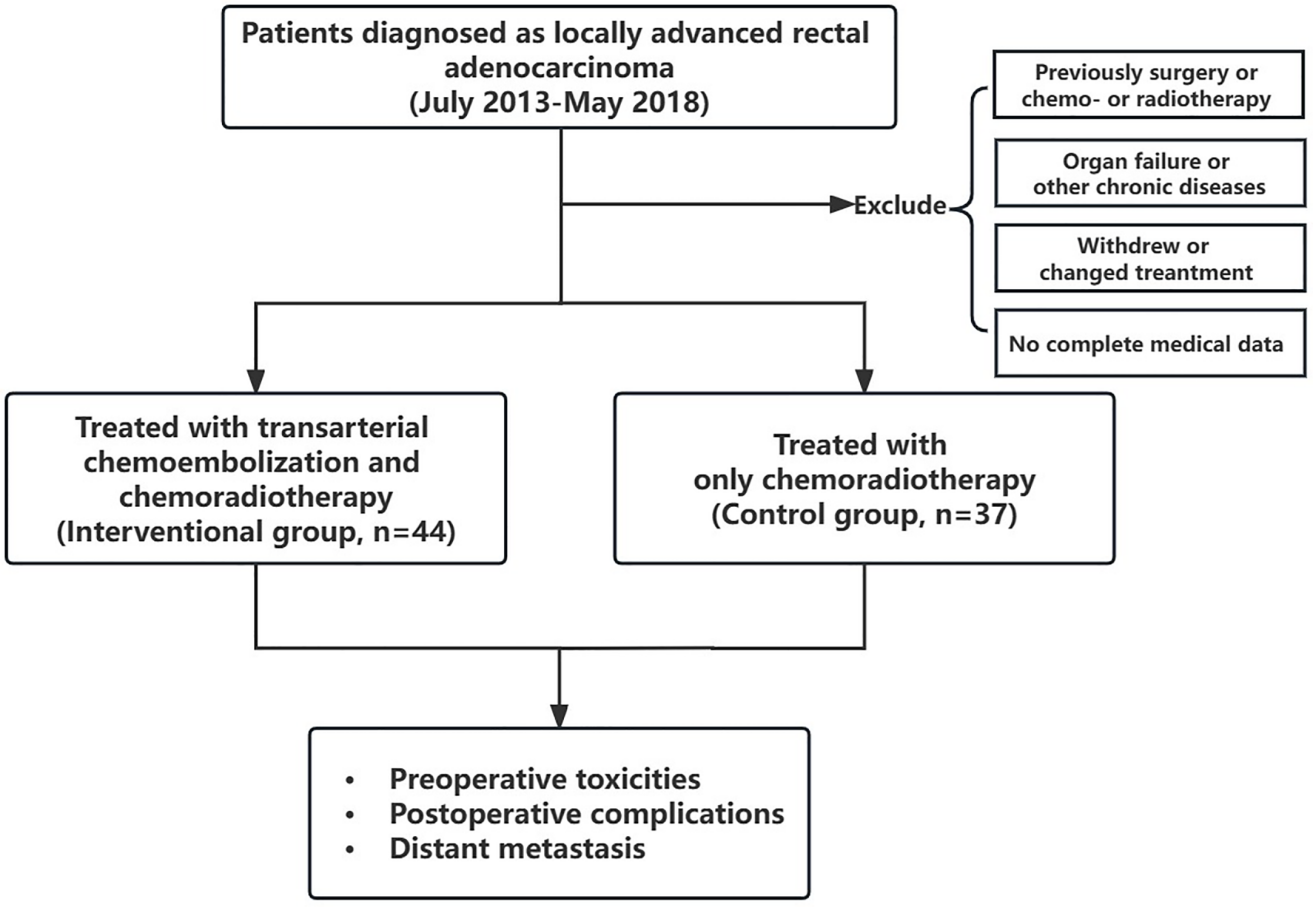
The flow diagram of this study.

Patients’ initial clinical information and follow-up data were collected. The initial baseline items included age, gender, tumor length, distance between the tumor and anal verge, carcinoembryonic antigen (CEA) level, lymphovascular invasion, and tumor staging pre- and post-treatment. Moreover, tumor regression grade (TRG) was assessed according to the American Joint Committee on Cancer (AJCC) criteria. The grade of TRG0 represents pathological complete remission. The preoperative toxicities and postoperative complications were also assessed. The 3-year DM status was followed up postoperatively in the two groups, and the number and rate of DM were recorded within 1, 2, and 3 years after surgery. The flow diagram of this study is shown in **Figure 2**.

### Statistical analysis

IBM SPSS 23.0 (IBM Corporation, Armonk, NY, USA) and GraphPad Prism 8.0.2 (GraphPad Software, San Diego, CA, USA) were used for statistical calculation and chart generation. Measurement data are represented by the mean ± *SD*, and then a *t*-test or Wilcoxon signed-rank test was performed according to whether the data were normally distributed. Enumeration data were analyzed by the chi-square test or Fisher’s exact test according to the sample size. A *p* < 0.05 indicated that the difference between the two groups was statistically significant.

## Results

### Patients’ demographic characteristics

There were no significant differences between the interventional group and the control group in terms of age, gender, tumor length, distance between the tumor and anal verge, CEA level, lymphovascular invasion, and tumor staging before treatment (*p* > 0.05). We found that the pathological T stage post-treatment in the interventional group was significantly reduced compared to that in the control group (*p* = 0.025). All patients successfully completed radical surgery, and R0 resection was performed among all patients except one with R1 resection in the control group. Also, at least four cycles (three months) of adjuvant chemotherapy were conducted in the two groups. The detailed data are shown in **Table 1**, suggesting that the two groups were statistically comparable.

**Table 1:** Patients’ characteristics of the two groups.

	Interventional group (*n* = 44)	Control group (*n* = 37)	*Z*/*χ* ^2^	*p*-value
Age (years)	56.6 ± 10.0	56.7 ± 10.1	−0.019	0.985
Gender (%)			0.063	0.802
Male	31 (70.5)	27 (73.0)		
Female	13 (29.5)	10 (27.0)		
Tumor length (cm)	3.8 ± 1.4	4.1 ± 1.9	−0.273	0.784
Tumor distance (cm)	5.8 ± 2.5	5.5 ± 2.2	−0.491	0.623
Baseline tumor staging (%)				
cT3/T4	36/8 (81.8/18.2)	28/9 (75.7/24.3)	0.457	0.499
N+/N-	31/13 (70.5/29.5)	24/13 (64.9/35.1)	0.288	0.591
II/III	13/31 (29.5/70.5)	13/24 (35.1/64.9)	0.288	0.591
Baseline CEA level (%)			0.359	0.549
≤5.0 ng/ml	29 (65.9)	22 (59.5)		
>5.0 ng/ml	15 (34.1)	15 (40.5)		
Pathological staging (%)				
ypT0/T1/T2/T3/T4	12/0/4/14/4 (27.3/0/31.8/9.1)	6/0/3/12/16 (16.2/0/32.4/43.2)	9.387	0.025
ypN+/N−	20/24 (45.5/54.5)	21/16 (56.8/43.2)	1.027	0.311
Tumor regression grade (TRG, %)			6.174	0.103
TRG0 (pCR)	14 (31.8)	7 (18.9)		
TRG1	13 (29.5)	6 (16.2)		
TRG2	12 (27.3)	14 (37.8)		
TRG3	5 (11.4)	10 (27.0)		
Quality of surgical resection (%)			/	0.457^a^
R0	44 (100)	36 (97.3)		
R1	0 (0)	1 (2.7)		
Lymphovascular invasion (%)			0.239	0.625
No	35 (79.5)	31 (83.8)		
Yes	9 (20.5)	6 (16.2)		
Adjuvant regimen (%)			0.132	0.716
CapeOX	22 (50.0)	20 (54.1)		
mFOLFOX6	22 (50.0)	17 (45.9)		
Time of adjuvant chemotherapy (months, %)			0.608	0.895
3	6 (13.6)	5 (13.5)		
4	10 (22.7)	6 (16.2)		
5	10 (22.7)	10 (27.0)		
6	18 (40.9)	16 (43.2)		

a Fisher’s exact test.

### Treatment-related toxicities and complications

During the pre- and postoperative process, we found that the related toxicities and complications were all endurable among all patients. In general, the occurrence of interventional chemoembolization-, chemoradiotherapy-, and surgery-related adverse events was similar between the interventional group and the control group. There were no statistically significant differences in toxicities and complications between the two groups (*p* > 0.05). The details of these treatment related adverse events are shown in **Table 2**.

**Table 2:** Treatment-related toxicities and complications between the interventional group and the control group.

Preoperative toxicities (%)	Interventional group (*n* = 44)	Control group (*n* = 37)	*χ* ^2^	*p*-value
Leukopenia	4 (9.1)	2 (5.4)	0.042	0.838^a^
Neutropenia	1 (2.3)	0 (0)	/	1.000^b^
Anemia	6 (13.6)	5 (13.5)	0.000	1.000^a^
Thrombocytopenia	2 (4.5)	1 (2.7)	0.000	1.000^a^
Nausea and vomiting	20 (45.5)	18 (48.6)	0.082	0.774
Fatigue	9 (20.5)	9 (24.3)	0.174	0.676
Diarrhea	10 (22.7)	7 (18.9)	0.176	0.675
Appetite loss	13 (29.5)	14 (37.8)	0.622	0.430
Radiation enteritis	9 (20.5)	5 (13.5)	0.279	0.597^a^
Postoperative complications (%)				
Incision infection	1 (2.3)	0 (0)	/	1.000^b^
Pelvic infection	1 (2.3)	1 (2.7)	0.000	1.000^a^
Intestinal obstruction	0 (0)	1 (2.7)	/	0.457^b^
Anastomotic leakage	1 (2.3)	1 (2.7)	0.000	1.000^a^
Urinary dysfunction	1 (2.3)	2 (5.4)	0.023	0.878^a^

a Continuous correction χ^2^ test;

b Fisher’s exact test.

### Distant metastasis

**Figure 3:**
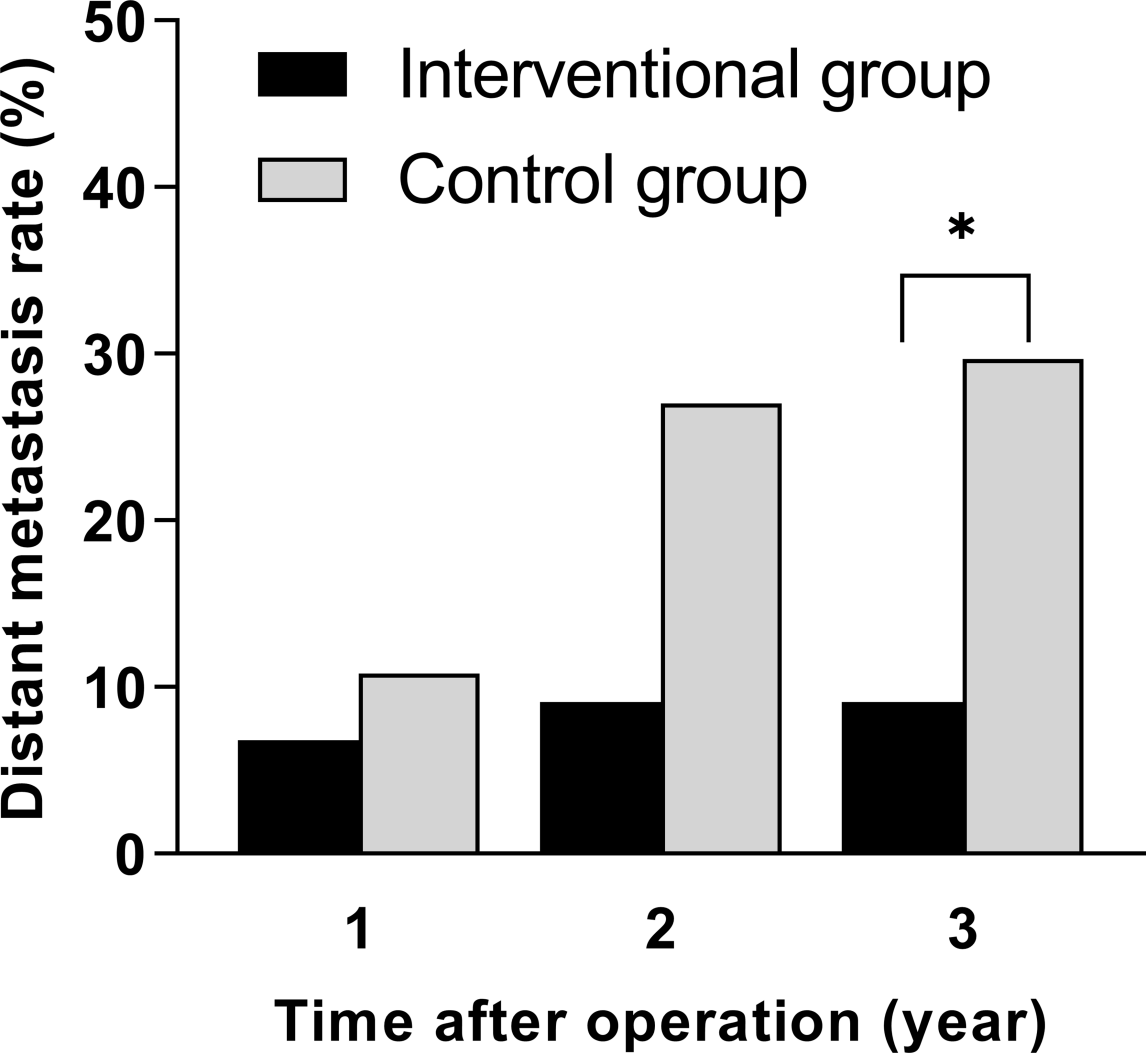
Comparison of distant metastasis rates within 1, 2, and 3 years after surgery between the interventional group and the control group (*p < 0.05).

**Figure 4:**
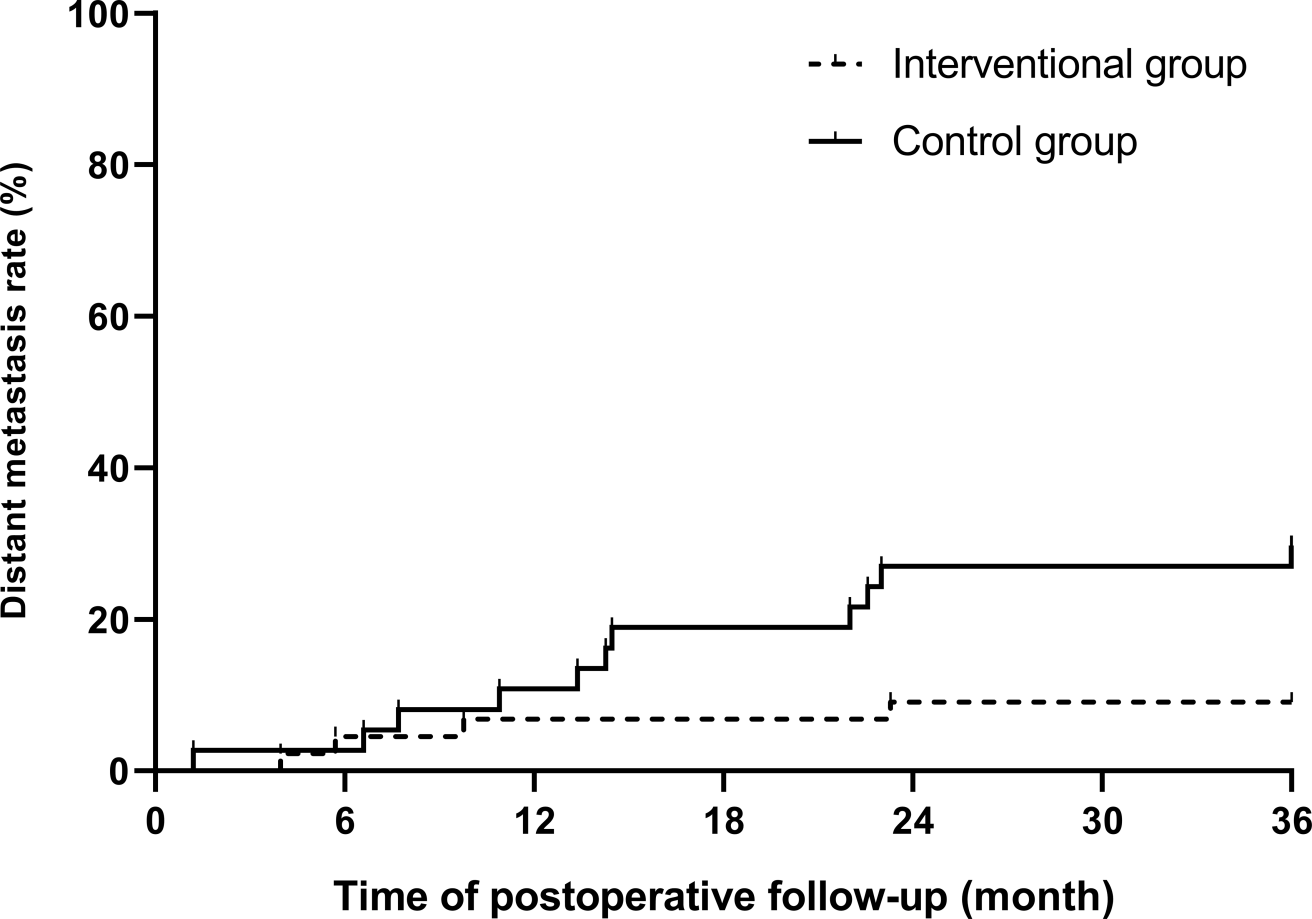
Kaplan–Meier curve of postoperative distant metastasis between the interventional group and the control group (*p* = 0.02).

During the 3-year follow-up after surgery, there were four cases of DM in the intervention group, including three cases of liver metastasis and one case of lung metastasis, and there were 11 cases of DM in the control group, including 5 cases of liver metastasis, 4 cases of lung metastasis, and 2 cases of bone metastasis. The 3-year DM rate in the intervention group was 9.1%, which was significantly lower than that in the control group (29.7%), and the difference was statistically significant (*p* = 0.036), as shown in **Table 3**. A bar chart is shown in **Figure 3** to compare the DM rates of the two groups within 1, 2, and 3 years after surgery. A Kaplan–Meier curve was also drawn in **Figure 4** to compare the DM of the two groups.

**Table 3:** Comparison of 3-year distant metastasis between the interventional group and the control group.

	Interventional group (*n* = 44)	Control group (*n* = 37)	*χ* ^2^	*p*-value
Liver metastasis (%)	3 (6.8)	5 (13.5)	0.400	0.527^a^
Lung metastasis (%)	1 (2.3)	4 (10.8)	1.270	0.260^a^
Bone metastasis (%)	0 (0)	2 (5.4)	/	0.206^b^
Metastasis within 1 year after surgery (%)	3 (6.8)	4 (10.8)	0.058	0.810^a^
Metastasis within 2 years after surgery (%)	4 (9.1)	10 (27.0)	3.355	0.067^a^
Metastasis within 3 years after surgery (%)	4 (9.1)	11 (29.7)	4.388	0.036^a^

a Continuous correction χ^2^ test;

b Fisher’s exact test.

## Discussion

In this study, all patients in the interventional group were successfully added and underwent interventional chemoembolization in preoperative treatments. Chemotherapeutic agents are mostly given intravenously; thus, local lesions and potential systemic micro-metastases are both taken into account. However, owing to the long time and cycle of administration, and the whole-body affection by these drugs, patients often experience a series of toxic reactions, resulting in delayed disease or even treatment failure. In addition, intravenously infused agents will bind to plasma proteins in the blood, leading to fast drug metabolism. Coupled with first-pass elimination, the remaining drugs may not be able to meet the minimum dose required to kill the tumor after reaching the regional tumor area (14). Therefore, it is difficult to prevent local tumor cells from spreading to the body, and more serious complications will occur if the dose of chemotherapeutic agents is blindly increasing. As the results suggested, no additional toxicities or complications were found in the interventional group compared to the control group. Furthermore, all of them underwent radical surgery as planned, and our preliminary study also confirmed that there was no negative impact on the safety of surgical operations and postoperative short-term outcomes (15).

In the first 2 years of follow-up, there was no significant difference in DM between the intervention group and the control group. After 3 years of follow-up, the 3-year DM rate of the intervention group was 9.1%, which was much lower than that of the control group (29.7%), and the difference was statistically significant (*p* < 0.05). The results indicated that the high-intensity killing effect of preoperative interventional chemoembolization on local tumor cells and subclinical metastases significantly reduced the probability of postoperative DM in LARC patients, and this advantage gradually appeared over time. During interventional chemotherapy and through the super-selection by microcatheters, high concentrations of drugs are directly perfused in a short period of time and accumulate in the tumor supplying arteries in the rectum, so that the primary tumor and its exfoliated tumor cells disseminated in surrounding tissues are continuously killed by the drugs. This more direct route enhances the killing effect of the agents on primary tumor cells. Because of this intra-arterial administration, drugs can directly enter the blood circulation starting from the rectal artery, accumulate in the portal vein, and then enter the liver. Compared with systemic intravenous chemotherapy, this method enables the first metabolism and accumulation of drugs to transfer to the liver, so it is more meaningful for preventing DM, especially liver metastasis (16). Recently, a randomized multi-center trial proved that preoperative hepatic and colorectal arterial chemotherapy is a safe method for colorectal cancer, which significantly improved the DFS rate after surgery (17). In this study, only the primary lesion feeding arteries were infused and embolized. After the end of infusion, embolization of the superior rectal arteries further disrupted the tumor blood supply, accelerated tumor necrosis, and prevented tumor cells from shedding into the systemic circulation, thereby reducing the probability of DM again. DM is the main cause of treatment failure in rectal cancer, and it is also the main cause of cancer-related death, which greatly reduces the survival time of patients with rectal cancer (18). Therefore, we have reason to believe that the improvement in the DM rate in the intervention group will have potential value for their longer-term survival benefit, which is also the direction of our future study.

There also remain limitations in this study. First, the sample size in this study was too small, so that propensity score matching could not be used to reach more convincing results. Second, the insufficient follow-up time is also a shortage, resulting in some unavoidable biases. Because of this, we did not collect enough statistics, such as the overall survival (OS) of these patients. For instance, a recent study indicated that among LARC patients, oxaliplatin-based adjuvant chemotherapy could only result in better DFS but not OS (19). In addition, the use of chemoembolization of tumor vessels can offset the anti-tumor effect of radiation. In this study, we did not take this factor into consideration. Another limitation concerns our insufficient pathological indicators such as intratumoral tumor budding (TB). In recent years, TB has been regarded as an important independent prognostic factor in LARC. Thus, more meaningful indicators related to prognosis need to be included in our subsequent studies. These limitations are inevitable for many reasons. Therefore, large, multi-center randomized controlled trials are still needed to verify the conclusions in this study.

In conclusion, preoperative regional transarterial chemoembolization combined with concurrent chemoradiotherapy may play an important role in reducing postoperative DM in patients with LARC. Further studies are needed to verify whether this method can improve other long-term prognosis indicators.

## Data availability statement

The raw data supporting the conclusions of this article will be made available by the authors, without undue reservation.

## Ethics statement

Written informed consent was obtained from the individual(s) for the publication of any potentially identifiable images or data included in this article.

## Author contributions

Conceptualization: C-XL. Methodology: C-XL and W-JM. Formal analysis and investigation: W-JM. Writing—original draft preparation: W-JM and C-HL. Writing—review and editing: R-JZ and C-XL. Funding acquisition: R-JZ and C-XL. Resources: C-XL. Supervision: C-XL. All authors contributed to the article and approved the submitted version.
